# Mapping research evidence on implementation of the WHO ‘best buys’ and other interventions for the prevention and control of non-communicable diseases in sub-Saharan Africa: a scoping review protocol

**DOI:** 10.1186/s13643-022-01992-7

**Published:** 2022-06-13

**Authors:** Adjei Kadiri, Monica Ansu-Mensah, Vitalis Bawontuo, Desmond Kuupiel

**Affiliations:** 1 Faculty of Health & Allied Sciences, Catholic University College of Ghana, Sunyani, Fiapre Ghana; 2 National Health Insurance Authority, Claims Processing Centre (CPC), Tamale, Northern Region Ghana; 3 Department of Public Health Medicine, School of Nursing and Public Health, University of KwaZulu-Natal, Durban, 4001 South Africa; 4 Department of Health Services Management and Administration, School of Business, SD Dombo University of Business and Integrated Development Studies (SDD-UBIDS), Wa, Ghana

**Keywords:** Non-communicable diseases, NCDs, ‘Best buys’, Unhealthy diet, Salt, Nutrition, Prevention and control, Sub-Saharan Africa

## Abstract

**Background:**

The rising burden of non-communicable diseases (NCDs) is a global health concern. To reduce the burden of morbidity, mortality and disability due to NCDs, the World Health Organization (WHO) developed ‘best buys’ and other interventions for the prevention and control of NCDs by member countries. However, their extent of implementation especially in sub-Saharan African countries (SSA) is not known. Therefore, this scoping review aims to map and describe research evidence on implementation of the WHO’s ‘best buys’ and other interventions for reducing unhealthy diets in SSA.

**Methods:**

This review will be guided by the enhanced version of Arksey and O’Malley’s framework and the recent Joanna Briggs Institute guidelines for scoping reviews. To identify the relevant published literature for this review, a comprehensive keyword search will be conducted in PubMed, SCOPUS, EBSCOhost (CINAHL, Health Resource and PsycINFO) and Cochrane Library from 2017 to 2021. Boolean terms (‘AND’ and ‘OR’), as well as Medical Subject Heading terms, will be included where essential. Government websites of SSA countries, the WHO’s website and Google Scholar will be consulted for grey literature such as governmental policies/strategies focus on reducing unhealthy diets. Moreover, the reference list of included evidence sources will be searched for additional literature. Two reviewers will independently screen the articles at the abstract and full-text screening phases guided by the review eligibility criteria. Also, all relevant data will be extracted independently by two reviewers, analysed thematically and the findings reported qualitatively.

**Discussion:**

The evidence produced by this review will help identify implementation and policy gaps to inform future implementation research/interventions studies using a variety of evidence-based strategies towards the prevention and control of NCDs due to unhealthy diets in the WHO Africa Region. Platforms such as peer review journals, policy briefs and conferences will be used to disseminate this review’s findings.

**Supplementary Information:**

The online version contains supplementary material available at 10.1186/s13643-022-01992-7.

## Background

Globally, the burden and threat of non-communicable diseases (NCDs) remain one of the major challenges of public health in the twenty-first century [1–4]. NCDs are chronic diseases or conditions that tend to have a long duration due to a combination of genetic, physiological, environmental and behavioural factors [5]. NCDs including heart disease, stroke, cancer, diabetes and chronic lung disease is ranked first among the causes of deaths and collectively responsible for over 71% of all deaths globally [5, 6]. Approximately two thirds of all NCD deaths and 82% of the 16 million people who died prematurely, or before reaching the age of 70 years, occur in low- and middle-income countries (LMICs) [5]. There is also a greater risk of premature death from NCDs in the WHO African region (22%) compared to the regions of the Americas (15%), European (17%) and the Western Pacific (16%) regions [4].

Worldwide, mortality due to NCDs is projected to reach 52 million by 2030 with 27% in the Africa region with the highest mortality occurring in sub-Saharan Africa (SSA) [7]. Moreover, deaths due to NCDs among populations between the ages of 10 and 60 years are predominant in SSA [8, 9], and also, there has been a substantial increase (67.0%) in disability-adjusted life years (DALYs) due to NCDs in SSA from 90.6 million in 1990 to 151.3 million in 2017 [10, 11].

The rising burden of NCDs in SSA is partially due to weak healthcare systems, resource constraints, low investment, limited infrastructure and capacity, combined with the already overwhelming burden of communicable, maternal, neonatal and nutritional (CMNN) diseases as well as low political commitment [6, 12, 13], Given this, there has been increased global advocacy to prioritise and address the growing burden of NCDs in SSA [14]. These culminated into the United Nations General Assembly declarations in 2011 (resolution A/RES/66/2), 2014 (A/RES/68/300) and 2018 (A/RES/73/2) to strengthen global and national responses to prevent and control NCDs [1, 15, 16]. Then, the WHO in 2017 recommended a set of interventions referred to as ‘Best buys’ which are considered cost-effective, affordable, feasible and evidence-based for implementation particularly in LMICs [17].

The ‘best buys’ are measures to reduce the four common risk factors of NCDs including tobacco use, unhealthy diet, physical inactivity and harmful use of alcohol in four disease areas of cardiovascular, diabetes, cancer and chronic respiratory diseases [2, 17]. Knowledge on the extent of adoption and implementation of the WHO ‘best buys’ is unknown, particularly in SSA, yet no previous scoping review has been previously conducted to examine the range of evidence and identify research gaps.

A scoping review is considered useful in mapping key concepts underpinning a research area, examining and clarifying broad areas to identify gaps in the evidence and reporting on the types of evidence that address and inform policy and practice in an area of study [18]. A scoping methodology is also considered a useful approach for preliminary mapping of evidence that will form the basis for determining the value of undertaking a full systematic review [18]. Therefore, we will conduct a scoping review to systematically map and describe research evidence on implementation of the WHO ‘best buys’ and other interventions for prevention and control of NCDs focusing on ‘unhealthy diet’ (Table 1) in SSA. We hope the results of this study will facilitate a better understanding of the practical application of the ‘best buys’ and other interventions to reduce the risk of NCDs and reveal knowledge gaps for future research in SSA. Moreover, this review may influence further research to inform policy as well as implementation research which may collectively contribute towards the attainment of the WHO 25 by 25 target and the Sustainable Development Goal target 3.4 which aims at reducing by 25% and one-third, the risk of premature mortality from non-communicable diseases by 2025 and 2030 respectively [2, 19, 20].

**Table 1:** WHO best buys and other recommended interventions for unhealthy diet

‘Best buys’: effective interventions with cost-effectiveness analysis (CEA) ≤ $100 per DALY averted in LMICs	Reduce salt intake through the reformulation of food products to contain less salt and the setting of target levels for the amount of salt in foods and meals
	Reduce salt intake through the establishment of a supportive environment in public institutions such as hospitals, schools, workplaces and nursing homes, to enable lower sodium options to be provided
	Reduce salt intake through a behaviour change communication and mass media campaign
	Reduce salt intake through the implementation of front-of-pack labelling
Effective interventions with CEA > $100 per DALY averted in LMICs	Eliminate industrial trans fats through the development of legislation to ban their use in the food chain
	Reduce sugar consumption through effective taxation on sugar-sweetened beverages
Other recommended interventions from WHO guidance (CEA not available)	Promote and support exclusive breastfeeding for the first 6 months of life, including promotion of breastfeeding
	Implement subsidies to increase the intake of fruits and vegetables
	Replace trans-fats and saturated fats with unsaturated fats, through reformulation, labelling, fiscal policies or agricultural policies
	Limiting portion and package size to reduce energy intake and the risk of overweight/obesity
	Implement nutrition education and counselling in different settings (for example, in preschools, schools, workplaces and hospitals) to increase the intake of fruits and vegetables
	Implement nutrition labelling to reduce total energy intake (kcal), sugars, sodium and fats
	Implement mass media campaign on healthy diets, including social marketing to reduce the intake of total fat, saturated fats, sugars and salt and promote the intake of fruits and vegetables

Source: WHO 2017 [17]

## Methods

This protocol was developing following the preferred reporting items for systematic reviews and meta-analysis extension for protocols (PRISMA-P) (Supplementary file 1). This scoping review will be conducted in keeping with Arksey and O’Malley’s methodological framework (identifying the research question; identifying relevant studies; study selection; charting the data; and collating, summarising and reporting results) [18], bearing in mind Levac et. al. recommendations [21] and the Joanna Briggs Institute checklist for scoping reviews [22].

### Identifying the research question

The main research question is as follows: for NCDs due to unhealthy diet such as type-2 diabetes, cardiovascular diseases, high cholesterol and some cancers, what research evidence exists on the adoption and implementation of the WHO’s ‘best buys’ and other interventions for their prevention and control in SSA since 2017 to date? Table 2 illustrates this review population, concept and context as part of the eligibility criteria. This scoping review sub-questions will be:

- What evidence exists on the implementation of the ‘best buys’ and other interventions to reduce the risk of NCDs due to unhealthy diets in SSA?
- What evidence exists on the barriers/challenges of implementing the ‘best buys’ and other interventions to reduce the risk of NCDs due to an unhealthy diet in SSA?

**Table 2:** PCC framework for defining the eligibility of the studies for the primary research question

Eligibility criteria	Include	Exclude
Population	All NCDs due to unhealthy diet by the general population such as type-2 diabetes, cardiovascular diseases, high cholesterol and some cancers	NCDs due to physical inactivity, tobacco use and harmful use of alcohol which has no linkage to unhealthy diet, e.g. lung cancer and chronic respiratory diseases
Concept	The WHO 2017 ‘best buys’ and other interventions for unhealthy diet	
Context	Prevention and/control of NCDs in SSA	
Setting	Countries in the WHO African Region [23]	Other WHO regions
Study design	Primary studies/grey literature (policy documents) that focus on the implementation of ‘best buys’ and other interventions for unhealthy diet.	Reviews that focus on the implementation of ‘best buys’ and other interventions for physical inactivity, tobacco use and harmful use of alcohol
Time frame	Publications/grey literature within the last 3 years	Publication/grey literature that existed before May 2017
Publication language	All international languages	

### Identifying relevant studies

The ‘best buys’ and other interventions for the prevention and control of NCDs were endorsed by the Seventieth World Health Assembly in May 2017. Given this, a thorough search for relevant published/grey literature will be conducted in PubMed, Google Scholar, SCOPUS, EBSCOhost (CINAHL, Health Resource, and PsycINFO), and Cochrane Library from June 2017 onwards to the search date. The World Health Organization and Governments/Ministries of Health websites will also be searched for relevant evidence sources. Moreover, the reference list of the included studies will be manually searched for relevant studies/grey literature. A comprehensive search strategy will be developed in consultation with an experienced librarian using a combination of the following keywords: ‘implementation’, ‘non-communicable diseases’, ‘chronic diseases’, ‘best buys’, ‘cost-effective, affordable and evidence-based interventions’, ‘salt intake’ food legislation’, ‘food labelling’, ‘fruits and vegetables’, ‘other interventions’, ‘prevention’, ‘control’, ‘unhealthy diet’, ‘sub-Saharan Africa’ and ‘all countries in the WHO Africa Region’. Boolean terms, ‘AND’ and ‘OR’, will be used to separate keywords. Medical Subject Heading (MeSH) terms and subject heading will be included where essential during the electronic database search. Study designs will be limited to primary studies and policy documents, and date (from June 2017 to 2021), but not language. Each search will be documented appropriately (Table 3), and Mendeley Desktop version 1.19.8 was used to compile and manage all references. The principal investigator (AK) will conduct the search, but a second reviewer will double-check for completeness.

**Table 3:** Pilot search in PubMed electronic database

Date	Database	Keywords	Search results
30 March 2021	PubMed	((((((((((((((((((((((((((((((((((((((((((((implementation) AND (best buys[MeSH Terms])) OR (best buys)) OR (cost-effective, affordable and evidence-based interventions[MeSH Terms])) OR (cost-effective, affordable and evidence-based interventions)) AND (cost-effective interventions[MeSH Terms])) OR (cost-effective interventions)) AND (affordable interventions[MeSH Terms])) OR (affordable interventions)) AND (evidence-based interventions[MeSH Terms])) OR (evidence-based interventions)) AND (prevention and control[MeSH Terms])) OR (prevention and control)) AND (prevention[MeSH Terms])) OR (prevention)) AND (control[MeSH Terms])) OR (control)) AND (noncommunicable diseases[MeSH Terms])) OR (non-communicable diseases[MeSH Terms])) OR (noncommunicable diseases)) OR (non-communicable diseases)) OR (chronic diseases[MeSH Terms])) AND (salt[MeSH Terms])) OR (salt)) AND (nutrition labelling[MeSH Terms])) OR (nutrition labelling)) OR (food labelling[MeSH Terms])) OR (food labelling)) AND (food legislation[MeSH Terms])) OR (food legislation)) AND (fruits and vegetables[MeSH Terms])) OR (fruits and vegetables)) AND (fruits[MeSH Terms])) OR (fruits)) AND (vegetables[MeSH Terms])) OR (vegetables)) AND (unhealthy diet[MeSH Terms])) OR (unhealthy diet)) AND (diet[MeSH Terms])) OR (diet)) AND (Sub-Saharan Africa)) OR (WHO African region)) OR (SSA) Filters: from 2017/6/1 - 2021/3/30	10,241

### Study selection

**Fig. 1:**
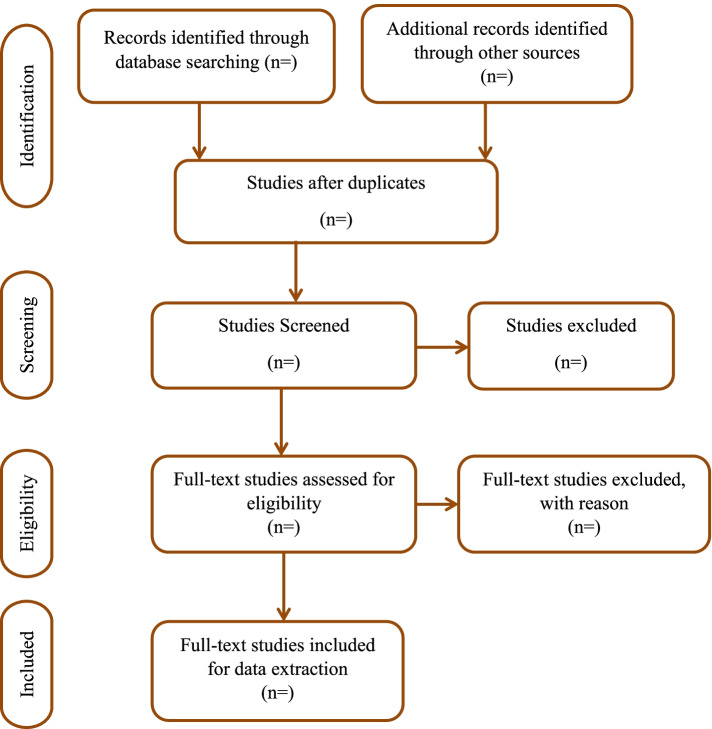
PRISMA 2009 flow diagram

Following deduplication of titles from the Mendeley desktop library created for this review, a screening tool will be developed using this study’s inclusion criteria in Google forms. The Google form will be shared with two members of the review team for pre-testing with a random sample of 10 titles and abstracts. The Google form will be amended if needed and, subsequently, use electronically for the study selection. The title and abstract screening (stage 1) will be performed by two reviewers (AK and MAM) independently guided by the inclusion and exclusion criteria. Then, the full text articles will be compiled and again screened (stage 2) by AK and MAM independently using the review eligibility criteria as a guide. A third reviewer will be consulted to resolve any discrepancies at both stages. The Catholic University library will be contacted to provide assistance in cases where a full article is inaccessible from the database or request will be made directly to authors of such articles via email for the full article. At least, the reviewers will make two attempts to reach the authors for full text articles if not found using other sources. The Preferred Reporting Items for Systematic Reviews and Meta-analysis (PRISMA) flow diagram [24] will be adopted to present the screening results of the study, as shown in Fig. 1.

### Charting the data

A data charting form will be designed for the extraction of all relevant data from the included studies. The form will contain the following headings: author(s) and year of publication, study title, objective/aim of the study, study design, country (study location), study setting (school, health facility, community, or others), study population, intervention(s), study results and ‘key findings relating to the implementation of the intervention for unhealthy diet’. To ensure the accuracy of the data extraction form, it will be pretested by two independent reviewers (AK and MAM) using a couple of the included studies and amended appropriately. AK will perform the data extraction, but two members of the review team will independently double-check the results extracted for completeness. The authors of the included evidence sources/studies will also be contacted to verify the results extracted.

### Collating, summarising, and reporting the results

Content thematic analysis approach [25] will be used to identify relevant themes and sub-themes to answer the review questions. The emerging themes will be structured around the four domains: effective interventions with cost-effectiveness analysis (CEA) less than or equivalent to hundred US dollars per DALYs averted in LMICs, effective interventions with CEA greater than hundred US dollars per DALYs averted in LMICs, other recommended interventions for the prevention and control of NCDs and barriers/challenges of implementing the ‘best buys’ and other interventions. The barriers/challenges will be identified using thematic content analysis. A qualitative approach will be used to report summaries of emerging key findings. Where appropriate, tables and figures will be used to represent this review’s findings, but tables and figures will also be used where possible. The Preferred Reporting Items for Systematic Reviews and Meta-analysis: Extension for Scoping Review (PRISMA-ScR) checklist will be followed to report this study.

## Discussion

This scoping review aims to identify and describe research evidence on the adoption and implementation of WHO ‘best buys’ and other interventions for the prevention and control of NCDs that relate to unhealthy diets in SSA. The review will have relevance to a variety of audiences including researchers, policymakers, NCD units and managers of various countries in SSA, donors and other interested bodies in understanding the extent of implementation of these ‘best buys’, as well as the barriers to implementation in SSA. Policymakers will also use this research for reflection and as a form of feedback on the progress of implementation of ‘best buys’ and other interventions for reducing the risk of NCDs due to unhealthy diet in SSA.

Evidence from WHO CEA demonstrates that investing in the implementation of the WHO ‘best buys’ and other interventions for NCDs prevention and control will not only improve health outcomes and save lives but can also improve a country’s economic productivity [13]. It is anticipated that the results of the proposed study will inform future research and reveal evidence-based information to address potential issues that may arise from implementation of the ‘best buys’ and other interventions for unhealthy diet in SSA. The proposed study will thus contribute to addressing implementation gaps, strengthen health systems and improve resource allocation as well as improve research in implementing the ‘best buys’ and other interventions to reduce NCDs in SSA.

Although there are four common risk factors of NCDs [2], due to resource and time constraints, this review will exclude evidence, policies and interventions relating to risk factors of tobacco use, physical inactivity and harmful use of alcohol.

Therefore, the results produced by this review will help identify implementation and policy gaps to inform future implementation research/interventions studies using a variety of evidence-based strategies towards the prevention and control of NCDs due to unhealthy diets in SSA.

## Supplementary Information

**Additional file 1.** PRISMA-P 2015 Checklist.

## Data Availability

All materials/data used for this review will be duly cited and presented in the form of references.

## Abbreviations

CEA: Cost-effectiveness analysis
CMNN: Communicable, maternal, neonatal and nutritional
DALYs: Disability-adjusted life years
LMICs: Low- and middle-income countries
NCDs: Non-communicable diseases
PRISMA: Preferred Reporting Items for Systematic Reviews and Meta-analysis
SSA: Sub-Sahara Africa
WHO: World Health Organization
